# Exponential growth of systematic reviews assessing artificial intelligence studies in medicine: challenges and opportunities

**DOI:** 10.1186/s13643-022-01984-7

**Published:** 2022-06-28

**Authors:** Thilo von Groote, Narges Ghoreishi, Maria Björklund, Christian Porschen, Livia Puljak

**Affiliations:** 1 Department of Anaesthesiology, Intensive Care and Pain Medicine, University Hospital Münster, Münster, Germany; 2 German Federal Institute for Risk Assessment, Berlin, Germany; 3 Library/ICT, Faculty of Medicine, Lund University, Lund, Sweden; 4 Center for Evidence-Based Medicine and Healthcare, Catholic University of Croatia, Zagreb, Croatia

**Keywords:** Artificial intelligence, Evidence-based medicine, Systematic reviews

## Abstract

The evidence-based medicine (EBM) movement is stepping up its efforts to assess medical artificial intelligence (AI) and data science studies. Since 2017, there has been a marked increase in the number of published systematic reviews that assess medical AI studies. Increasingly, data from observational studies are used in systematic reviews of medical AI studies. Assessment of risk of bias is especially important in medical AI studies to detect possible “AI bias”.

**Supplementary Information:**

The online version contains supplementary material available at 10.1186/s13643-022-01984-7.

Dear Editor,

With digitalization and enhanced computing power, the scientific community is amassing data at an unprecedented rate. With this big data, clinicians and biomedical researchers collaborate with computer scientists to use artificial intelligence (AI) to detect signals from noise [1]. AI and data science are expected to contribute to significant improvements in healthcare and medicine [2]. Therefore, it appears essential to synthesize evidence from medical AI studies and assess the quality of these new data-driven interventions and tools.

Among various types of evidence, systematic reviews and meta-analyses are the standard for guideline development and guide researchers, clinicians, and policymakers alike [3]. Furthermore, increased quality of evidence-synthesis may support patients and physicians to trust the AI applications and their adoption in the healthcare sector.

## The number of systematic reviews assessing studies on medical AI is growing rapidly

**Fig. 1:**
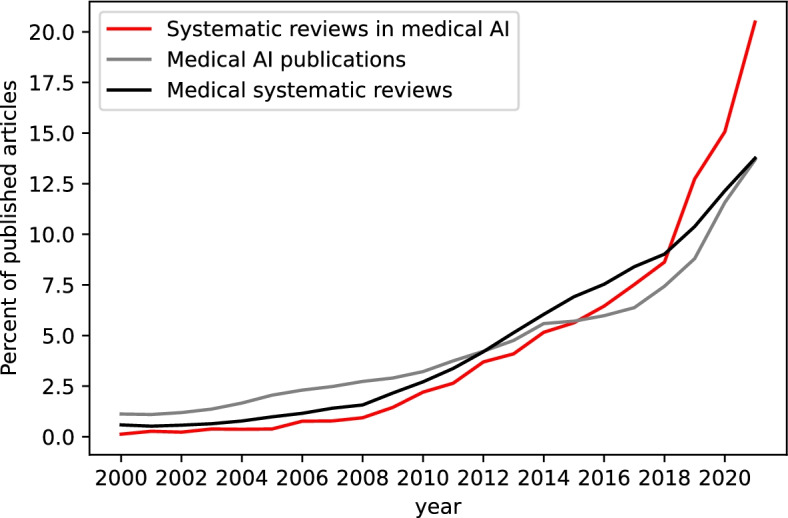
Medical articles on artificial intelligence (AI), systematic reviews overall, and systematic reviews specifically investigating medical AI studies as a percentage of published articles overall; indexed per year in PubMed and EMBASE, from 2000 to 2021. Supplementary file 1 reports search strategies and software used to retrieve and analyze these records. Supplementary file 2 reports tabular results of the search

Since its beginning in the 1980s, the EBM movement has fostered the development of evidence syntheses, which is reflected in the rapidly growing number of systematic reviews published each year (see the black line, Fig. 1). Starting some years later, the number of medical AI studies has been proliferating similarly with a rapid pace since 2000 and a marked increase from 2017 onwards (see the gray line, Fig. 1). However, we could not find reports in the literature about the number and characteristics of systematic reviews which include medical AI studies. To assess the number of such studies and whether their number was growing in line with the medical AI studies, we performed an extensive literature search that analyzed first the number of all medical publications in PubMed and EMBASE. Then, both absolute numbers and percentages were compared in the three groups: systematic reviews overall, medical AI studies overall, and systematic reviews with a medical AI topic (Fig. 1). Percentages are shown in Fig. 1 for ease of comparison.

Compared to the overall number of systematic reviews published and to the immense growth in medical AI research, the proportion of systematic reviews specifically investigating medical AI studies has been keeping up in rate and even exceeds the pace of the former two in recent years (See the red line, Fig. 1).

## The importance of systematic reviews on medical AI studies: challenges and opportunities

**Fig. 2:**
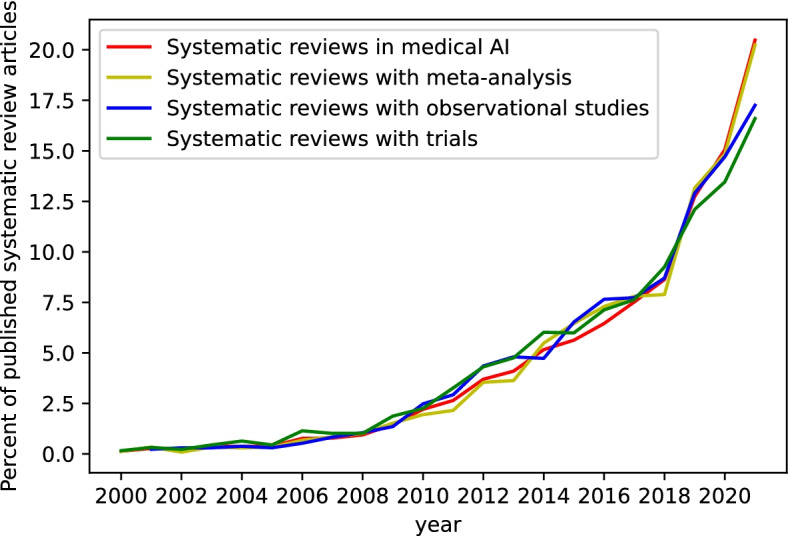
Medical AI systematic reviews in terms of content: containing a meta-analysis, containing observational studies, and containing randomized controlled studies as a percentage of published articles overall; indexed per year in PubMed and EMBASE, from 2000 to 2021

Systematic reviews on medical AI studies are of high importance to guide the implementation of AI tools in the healthcare sector and to provide an overview of AI-provided evidence for medical researchers, physicians, and patients. However, there are multiple challenges associated with this type of evidence synthesis. One is the potential risk of bias [4]. Especially in machine-learning models, the training population has significant implications for an algorithms’ performance, generalizability, and its equity or discrimination, with a significant risk for the so-called AI bias [4–6]. The risk of selection bias with AI data is an important methodological consideration. The growing number of observational studies in medical AI systematic reviews (Fig. 2) calls for more attention to bias due to the non-randomized format of these studies. In addition, medical AI methods tend to require big data and, as a result, are heavily based on secondary data (data that is not collected for the purpose of the research), such as electronic medical records [7].

Since its inception, the EBM movement has played a vital role in challenging bias and scrutinizing the scientific evidence from clinical trials and biomedical studies. Given the special importance of systematic reviews for the assessment of the evidence from medical AI studies, the rapid growth in the number of such reviews must be acknowledged as a positive development. In our opinion, this field holds much potential and room for further quality improvement. Therefore, a focus on investment in and development of adequate training and tools for EBM researchers to assess medical AI studies through high-quality systematic reviews would be worthwhile. Now is the time to intensify these efforts.

## Supplementary Information

**Additional file 1.** Search strategy to identify publications on AI/ML.

**Additional file 2.**

## Data Availability

The search strategy is available in the supplementary files. Obtained data are available upon request.

## Abbreviations

AI: Artificial intelligence
EBM: Evidence-based medicine
EMBASE: Excerpta Medica database
