# Supplementary material for: Exponential growth of systematic reviews assessing artificial intelligence studies in medicine: challenges and opportunities
Source: Syst Rev. 2022 Jun 28;11:132. doi: 10.1186/s13643-022-01984-7 (PMC9238033; doi:10.1186/s13643-022-01984-7)
Supplement: Supplementary file 1 — Additional file 1. Search strategy to identify publications on AI/ML. [file 13643_2022_1984_MOESM1_ESM.docx]

**Supplementary file**

**Search strategy to identify publications on AI/ML**

Date of search 2021, May 9

**PubMed**

#1 ("artificial intelligence"[MeSH Terms]) OR ("multicriteria decision analysis"[Title/Abstract]) OR ("automated reasoning"[Title/Abstract]) OR ("ambient intelligence"[Title/Abstract]) OR (artificial intelligence[Title/Abstract]) OR AI [Title/Abstract] OR ("expert systems"[Title/Abstract]) OR ("fuzzy logic"[Title/Abstract]) OR ("knowledge bases"[Title/Abstract]) OR ("natural language processing"[Title/Abstract])

154943 records

#2 (machine learning[MeSH Terms]) OR (machine learning[Title/Abstract] OR ML[Title/Abstract])

386247 records

#3 (systematic review[Title/Abstract]) OR (systematic review[Publication Type])

211432 records

#4 (robotic*[Title/Abstract]) OR ("robotics"[MeSH Terms])

48405 records

#5 #1 OR #2

509069 records

#6 #5 AND #3

3127 records

#7 #6 NOT #4

2752 records

#8 #7 NOT animal

2666 records

#9 #8 AND Publication date limitation 2000-20211231

2664 records

Number of original articles as comparison:

348674

(Articles on AI/ML covered by searches above, excluding robotics, animals, published 2000-2021)

**Embase (Elsevier)**

#1 'artificial intelligence'/exp OR 'artificial intelligence':ab,ti OR ai:ab,ti OR 'expert systems':ab,ti OR 'fuzzy logic':ab,ti OR 'knowledge bases':ab,ti OR 'natural language processing':ab,ti OR 'computer neural networks':ab,ti OR 'multicriteria decision analysis':ab,ti OR 'automated reasoning':ti,ab OR 'ambient intelligence':ti,ab

92395 records

#2 'machine learning'/exp OR 'machine learning':ab,ti OR ml:ab,ti

1428199 records

#3 'systematic review':it OR 'systematic review':ab,ti

225401 records

#4 'robotics'/exp OR robotic*:ab,ti

76988 records

#5 #1 OR #2

1479950 records

#6 #5 AND #3

4604 records

#7 #6 NOT #4

4514 records

#8 #7 NOT animal

4391 records

#9 #8 AND Publication date limitation 2000-20211231

4381 records

#10 #8 AND [embase]/lim NOT ([embase]/lim AND [medline]/lim)

1549 records

Number of original articles as comparison:

475112

(Articles on AI/ML covered by searches above, excluding robotics, animals, excluding conference abstracts, and including Embase unique records only, published 2000-2021)

4213 records, 4013 records after deduplication in EndNote
